# Crystal structure of 2-(2,4-di­chloro­phen­yl)-4-hydroxy-9-phenyl­sulfonyl-9*H*-carbazole-3-carbaldehyde

**DOI:** 10.1107/S1600536814024064

**Published:** 2014-11-08

**Authors:** M. Umadevi, B. M. Ramalingam, R. Yamuna, A. K. Mohanakrishnan, G. Chakkaravarthi

**Affiliations:** a Research and Development Centre, Bharathiyar University, India; b Department of Chemistry, Pallavan College of Engineering, Kanchipuram, Tamilnadu, India; c Department of Organic Chemistry, University of Madras, Guindy Campus, Chennai 600 025, India; d Department of Sciences, Chemistry and Materials Research Laboratory, Amrita Vishwa Vidyapeetham University, Ettimadai, Coimbatore 641 112, India; e Department of Physics, CPCL Polytechnic College, Chennai 600 068, India

**Keywords:** crystal structure, carbazole derivative, hydrogen bonding

## Abstract

In the title compound, C_25_H_15_Cl_2_NO_4_S, the di­chloro­phenyl ring is twisted by 68.69 (11)° from the mean plane of the carbazole ring system [r.m.s. deviation = 0.084 (2)°]. The hy­droxy group is involved in an intra­molecular O—H⋯O hydrogen bond, which generates an *S*(6) graph-set motif. In the crystal, pairs of C—H⋯Cl hydrogen bonds link mol­ecules into inversion dimers with an *R* ^2^_2_(26) motif. Weak C—H⋯O inter­actions further link these dimers into ribbons propagating in [100].

## Abstract 2

The hy­droxy group in this carbazole derivative is involved in an intra­molecular O—H⋯O hydrogen bond, which generates an *S*(6) graph-set motif. In the crystal, pairs of C—H⋯Cl hydrogen bonds link mol­ecules into inversion dimers with an *R* ^2^_2_(26) motif. Weak C—H⋯O inter­actions further link these dimers into ribbons propagating in [100].

## Chemical context   

**Figure 1:**
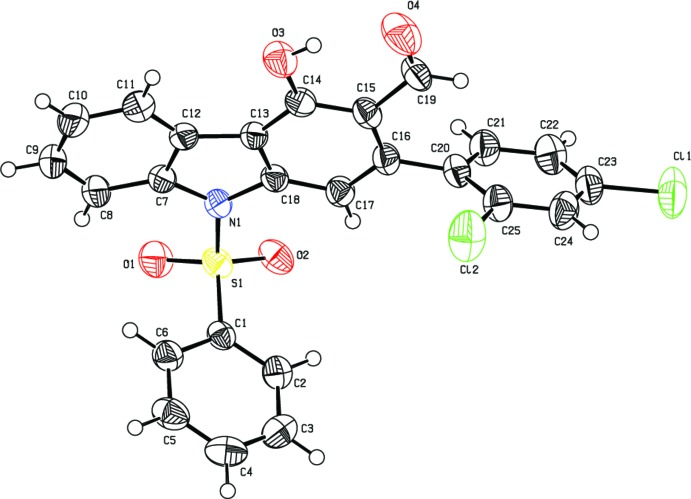
The mol­ecular structure of (I) showing the atomic labelling scheme and 50% probability displacement ellipsoids.

In continuation of our studies of carbazole derivatives, which are found to possess various biological activities, such as anti-oxidative (Tachibana *et al.*, 2001 ▶), anti-inflammatory and anti­mutagenic (Ramsewak *et al.*, 1999 ▶), anti­biotic, anti­fungal and cytotoxic (Chakraborty *et al.*, 1965 ▶, 1978 ▶), we report herein on the synthesis and crystal structure of the title compound (I) (Fig. 1 ▶).
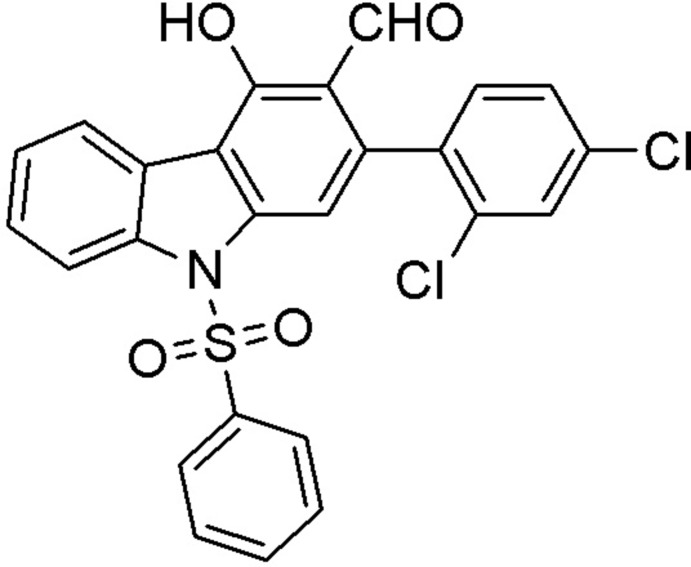


## Structural commentary   

The geometric parameters of (I) agree well with those reported for related structures [Chakkaravarthi *et al.* 2008 ▶, 2009 ▶]. The C1–C6 phenyl ring makes a dihedral angle of 79.76 (11)° with the carbazole ring system (C7–C18/N1). The di­chloro­phenyl ring (C21–C25) is twisted by 68.69 (11)° from the mean plane of the carbazole ring system and inclined at an angle of 32.22 (13)° to the phenyl ring.

Atom S1 has a distorted tetra­hedral configuration. The widening of angle O1—S1—O2 [120.49 (11)°] and narrowing of angle N1—S1—C1 [105.36 (10)°] from the ideal tetra­hedral values are attributed to the Thorpe–Ingold effect (Bassindale, 1984 ▶). As a result of the electron–withdrawing character of the phenyl­sulfonyl group, the bond lengths N1—C7 [1.431 (3) Å] and N1—C18 [1.414 (3) Å] in the mol­ecule are longer than the mean value of 1.355 (14) Å (Allen *et al.*, 1987 ▶; Groom & Allen *et al.*, 2014 ▶).

## Supra­molecular features   

**Figure 2:**
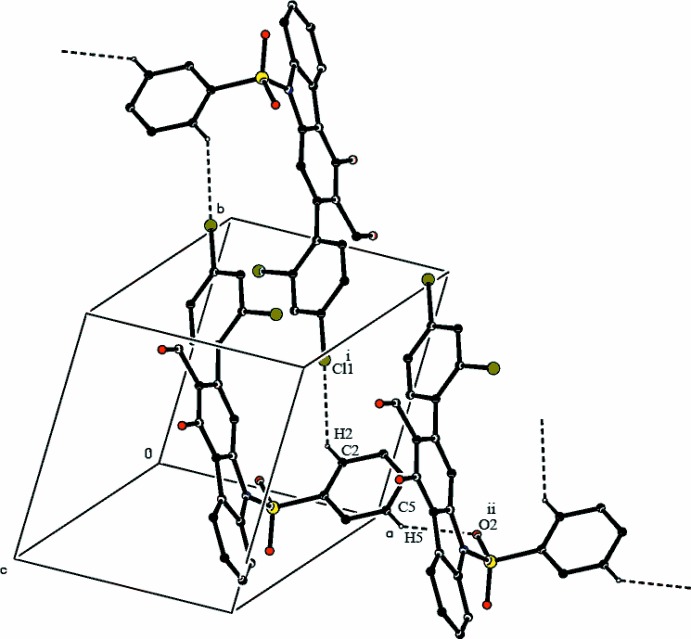
A portion of the crystal packing of (I) showing the hydrogen-bonded (dashed lines) ribbon [symmetry codes: (i) 1-*x*, 2-*y*, 1-*z*; (ii) 1 + *x*, *y*, *z*].

The hy­droxy group is involved in an intra­molecular O—H⋯O hydrogen bond (Table 1 ▶), which generates an *S*(6) graph-set motif. In the crystal, pairs of C—H⋯Cl hydrogen bonds link mol­ecules into inversion dimers with an 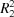
(26) motif (Bernstein *et al.*, 1995 ▶), and weak C—H⋯O inter­actions further link these dimers into ribbons propagating in [100] (Table 1 ▶ and Fig. 2 ▶) .

## Synthesis and crystallization   

Enamine 16 g (500 mg, 0.95 mmol) was reacted with CuBr_2_ (212 mg, 0.95 mmol) in dry DMF (20 ml) at reflux for 1 h under N_2_. The reaction mass was poured over crushed ice (50 ml) containing concentrated HCl (1 ml). The precipitated solid was filtered, washed with water and air-dried to obtain the crude compound, which was purified by flash column chromatography on silica gel (230–420 mesh, *n*-hexa­ne/ethyl acetate, 7:3) to afford 17 g as pale-yellow crystals suitable for X-ray analysis. Yield: 368 mg (78%); m.p.: 461–463 K.

## Refinement   

Crystal data, data collection and structure refinement details are summarized in Table 2 ▶. The hy­droxy H atom was located in a difference Fourier map and refined isotropically with a distance restraint of O—H = 0.82 (1) Å. All other H atoms were positioned geometrically and refined using a riding model, with C—H = 0.93 Å and *U*_iso_(H) = 1.2*U*_eq_(C). The components of the anisotropic displacement parameters in the direction of the bond between O4 and C19 were restrained to be equal within an effective standard deviation of 0.001 using the DELU command in *SHELXL97* (Sheldrick, 2008 ▶).

## Supplementary Material

Crystal structure: contains datablock(s) global, I. DOI: 10.1107/S1600536814024064/cv5474sup1.cif

Structure factors: contains datablock(s) I. DOI: 10.1107/S1600536814024064/cv5474Isup2.hkl

Supporting information file. DOI: 10.1107/S1600536814024064/cv5474Isup3.cml

CCDC reference: 1032055

Additional supporting information:  crystallographic information; 3D view; checkCIF report

## Figures and Tables

**Table 1:** Hydrogen-bond geometry (, )

*D*H*A*	*D*H	H*A*	*D* *A*	*D*H*A*
O3H3*A*O4	0.83(1)	1.81(2)	2.563(3)	151(3)
C2H2Cl1^i^	0.93	2.81	3.412(2)	123
C5H5O2^ii^	0.93	2.49	3.184(3)	131

Symmetry codes: (i) 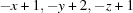; (ii) 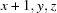.

**Table 2:** Experimental details

Crystal data	
Chemical formula	C_25_H_15_Cl_2_NO_4_S
*M*_r_	496.34
Crystal system, space group	Triclinic, *P* 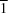
Temperature (K)	295
*a*, *b*, *c* ()	8.0688(4), 9.9086(5), 14.4041(8)
, , ()	75.297(3), 80.604(2), 74.306(3)
*V* (^3^)	1066.83(10)
*Z*	2
Radiation type	Mo *K*
(mm^1^)	0.44
Crystal size (mm)	0.28 0.24 0.18

Data collection	
Diffractometer	Bruker Kappa APEXII CCD
Absorption correction	Multi-scan (*SADABS*; Sheldrick, 1996 ▶)
*T*_min_, *T*_max_	0.887, 0.925
No. of measured, independent and observed [*I* > 2(*I*)] reflections	35959, 6221, 3582
*R*_int_	0.044
(sin /)_max_ (^1^)	0.735

Refinement	
*R*[*F* ^2^ > 2(*F* ^2^)], *wR*(*F* ^2^), *S*	0.048, 0.126, 1.02
No. of reflections	6221
No. of parameters	302
No. of restraints	2
H-atom treatment	H atoms treated by a mixture of independent and constrained refinement
_max_, _min_ (e ^3^)	0.44, 0.48

Computer programs: *APEX2* and *SAINT* (Bruker, 2004 ▶), *SHELXS97* and *SHELXL97* (Sheldrick, 2008 ▶) and *PLATON* (Spek, 2009 ▶).

## supplementary crystallographic information

### Crystal data

**Table:**

C_25_H_15_Cl_2_NO_4_S	*Z* = 2
*M**_r_* = 496.34	*F*(000) = 508
Triclinic, *P*1	*D*_x_ = 1.545 Mg m^−^^3^
Hall symbol: -P 1	Mo *K*α radiation, λ = 0.71073 Å
*a* = 8.0688 (4) Å	Cell parameters from 9493 reflections
*b* = 9.9086 (5) Å	θ = 2.2–31.5°
*c* = 14.4041 (8) Å	µ = 0.44 mm^−^^1^
α = 75.297 (3)°	*T* = 295 K
β = 80.604 (2)°	Block, pale yellow
γ = 74.306 (3)°	0.28 × 0.24 × 0.18 mm
*V* = 1066.83 (10) Å^3^	

### Data collection

**Table:**

Bruker Kappa APEXII CCD diffractometer	6221 independent reflections
Radiation source: fine-focus sealed tube	3582 reflections with *I* > 2σ(*I*)
Graphite monochromator	*R*_int_ = 0.044
ω and φ scan	θ_max_ = 31.5°, θ_min_ = 2.2°
Absorption correction: multi-scan (*SADABS*; Sheldrick, 1996)	*h* = −11→11
*T*_min_ = 0.887, *T*_max_ = 0.925	*k* = −14→13
35959 measured reflections	*l* = −19→21

### Refinement

**Table:**

Refinement on *F*^2^	Primary atom site location: structure-invariant direct methods
Least-squares matrix: full	Secondary atom site location: difference Fourier map
*R*[*F*^2^ > 2σ(*F*^2^)] = 0.048	Hydrogen site location: inferred from neighbouring sites
*wR*(*F*^2^) = 0.126	H atoms treated by a mixture of independent and constrained refinement
*S* = 1.02	*w* = 1/[σ^2^(*F*_o_^2^) + (0.0395*P*)^2^ + 0.6935*P*] where *P* = (*F*_o_^2^ + 2*F*_c_^2^)/3
6221 reflections	(Δ/σ)_max_ < 0.001
302 parameters	Δρ_max_ = 0.44 e Å^−^^3^
2 restraints	Δρ_min_ = −0.48 e Å^−^^3^

### Special details

**Table:**

Geometry. All esds (except the esd in the dihedral angle between two l.s. planes) are estimated using the full covariance matrix. The cell esds are taken into account individually in the estimation of esds in distances, angles and torsion angles; correlations between esds in cell parameters are only used when they are defined by crystal symmetry. An approximate (isotropic) treatment of cell esds is used for estimating esds involving l.s. planes.
Refinement. Refinement of F^2^ against ALL reflections. The weighted R-factor wR and goodness of fit S are based on F^2^, conventional R-factors R are based on F, with F set to zero for negative F^2^. The threshold expression of F^2^ > 2sigma(F^2^) is used only for calculating R-factors(gt) etc. and is not relevant to the choice of reflections for refinement. R-factors based on F^2^ are statistically about twice as large as those based on F, and R- factors based on ALL data will be even larger.

### Fractional atomic coordinates and isotropic or equivalent isotropic displacement parameters (Å^2^)

**Table:**

	*x*	*y*	*z*	*U*_iso_*/*U*_eq_	
C1	1.0997 (3)	0.3850 (2)	0.70308 (15)	0.0345 (5)	
C2	1.0940 (3)	0.4840 (3)	0.61674 (17)	0.0471 (6)	
H2	0.9939	0.5159	0.5854	0.057*	
C3	1.2388 (4)	0.5356 (3)	0.5769 (2)	0.0582 (7)	
H3	1.2371	0.6018	0.5181	0.070*	
C4	1.3848 (3)	0.4886 (3)	0.6244 (2)	0.0551 (7)	
H4	1.4821	0.5234	0.5974	0.066*	
C5	1.3893 (3)	0.3910 (3)	0.71103 (19)	0.0524 (6)	
H5	1.4889	0.3607	0.7428	0.063*	
C6	1.2472 (3)	0.3379 (3)	0.75090 (17)	0.0445 (5)	
H6	1.2499	0.2710	0.8094	0.053*	
C7	0.8949 (3)	0.3246 (2)	0.94145 (16)	0.0384 (5)	
C8	1.0086 (3)	0.1961 (2)	0.97765 (19)	0.0483 (6)	
H8	1.0604	0.1300	0.9395	0.058*	
C9	1.0419 (3)	0.1703 (3)	1.07175 (19)	0.0530 (6)	
H9	1.1187	0.0853	1.0975	0.064*	
C10	0.9647 (3)	0.2669 (3)	1.12916 (19)	0.0557 (7)	
H10	0.9915	0.2468	1.1924	0.067*	
C11	0.8483 (3)	0.3927 (3)	1.09402 (17)	0.0487 (6)	
H11	0.7938	0.4565	1.1334	0.058*	
C12	0.8138 (3)	0.4224 (2)	0.99882 (16)	0.0376 (5)	
C13	0.7085 (3)	0.5439 (2)	0.93908 (15)	0.0349 (5)	
C14	0.5999 (3)	0.6708 (2)	0.95850 (16)	0.0387 (5)	
C15	0.5169 (3)	0.7739 (2)	0.88353 (17)	0.0404 (5)	
C16	0.5471 (3)	0.7497 (2)	0.78918 (17)	0.0391 (5)	
C17	0.6519 (3)	0.6224 (2)	0.76991 (16)	0.0392 (5)	
H17	0.6704	0.6054	0.7080	0.047*	
C18	0.7289 (3)	0.5206 (2)	0.84630 (16)	0.0350 (5)	
C19	0.3929 (3)	0.9054 (3)	0.9062 (2)	0.0491 (6)	
H19	0.3362	0.9730	0.8567	0.059*	
C20	0.4596 (3)	0.8596 (2)	0.70952 (17)	0.0415 (5)	
C21	0.3271 (3)	0.8330 (3)	0.67214 (19)	0.0500 (6)	
H21	0.2988	0.7447	0.6954	0.060*	
C22	0.2357 (4)	0.9332 (3)	0.6016 (2)	0.0578 (7)	
H22	0.1467	0.9132	0.5777	0.069*	
C23	0.2786 (4)	1.0625 (3)	0.56730 (18)	0.0529 (6)	
C24	0.4114 (4)	1.0926 (2)	0.60002 (19)	0.0534 (6)	
H24	0.4407	1.1803	0.5752	0.064*	
C25	0.5009 (3)	0.9905 (2)	0.67056 (18)	0.0478 (6)	
N1	0.8388 (2)	0.38241 (18)	0.84706 (13)	0.0383 (4)	
O1	0.9772 (2)	0.16411 (16)	0.78521 (13)	0.0539 (5)	
O2	0.7943 (2)	0.37100 (18)	0.68385 (12)	0.0480 (4)	
O3	0.5763 (2)	0.6893 (2)	1.04911 (13)	0.0545 (5)	
O4	0.3634 (3)	0.9274 (2)	0.98621 (16)	0.0726 (6)	
S1	0.92029 (7)	0.31431 (6)	0.75094 (4)	0.03877 (15)	
Cl1	0.16251 (12)	1.19156 (9)	0.48058 (6)	0.0826 (3)	
Cl2	0.66928 (11)	1.03092 (8)	0.71005 (7)	0.0781 (3)	
H3A	0.512 (4)	0.7704 (18)	1.047 (2)	0.084 (11)*	

### Atomic displacement parameters (Å^2^)

**Table:**

	*U*^11^	*U*^22^	*U*^33^	*U*^12^	*U*^13^	*U*^23^
C1	0.0343 (11)	0.0338 (10)	0.0355 (12)	−0.0022 (9)	−0.0034 (9)	−0.0143 (9)
C2	0.0419 (13)	0.0501 (14)	0.0455 (14)	−0.0059 (11)	−0.0096 (11)	−0.0059 (11)
C3	0.0609 (17)	0.0609 (16)	0.0473 (15)	−0.0190 (14)	−0.0006 (13)	−0.0007 (12)
C4	0.0466 (15)	0.0690 (17)	0.0585 (17)	−0.0252 (13)	0.0080 (12)	−0.0257 (14)
C5	0.0367 (13)	0.0736 (18)	0.0516 (16)	−0.0115 (12)	−0.0065 (11)	−0.0229 (13)
C6	0.0376 (12)	0.0531 (14)	0.0401 (13)	−0.0060 (11)	−0.0060 (10)	−0.0093 (11)
C7	0.0346 (11)	0.0329 (11)	0.0424 (13)	−0.0066 (9)	0.0005 (9)	−0.0035 (9)
C8	0.0454 (14)	0.0340 (12)	0.0541 (15)	−0.0009 (10)	0.0008 (11)	−0.0024 (11)
C9	0.0464 (14)	0.0433 (13)	0.0551 (16)	−0.0019 (11)	−0.0061 (12)	0.0055 (12)
C10	0.0512 (15)	0.0641 (17)	0.0435 (14)	−0.0102 (13)	−0.0095 (12)	0.0017 (13)
C11	0.0479 (14)	0.0527 (14)	0.0409 (14)	−0.0082 (11)	−0.0015 (11)	−0.0082 (11)
C12	0.0344 (11)	0.0346 (11)	0.0404 (12)	−0.0089 (9)	0.0014 (9)	−0.0047 (9)
C13	0.0333 (11)	0.0311 (10)	0.0380 (12)	−0.0068 (8)	−0.0012 (9)	−0.0058 (9)
C14	0.0378 (12)	0.0394 (12)	0.0401 (12)	−0.0083 (9)	−0.0018 (10)	−0.0134 (10)
C15	0.0358 (12)	0.0334 (11)	0.0503 (14)	−0.0030 (9)	−0.0047 (10)	−0.0119 (10)
C16	0.0381 (12)	0.0313 (11)	0.0454 (13)	−0.0041 (9)	−0.0075 (10)	−0.0066 (9)
C17	0.0416 (12)	0.0350 (11)	0.0392 (12)	−0.0055 (9)	−0.0039 (10)	−0.0091 (9)
C18	0.0320 (11)	0.0286 (10)	0.0418 (12)	−0.0059 (8)	0.0001 (9)	−0.0070 (9)
C19	0.0362 (12)	0.0467 (13)	0.0581 (13)	0.0058 (10)	−0.0078 (11)	−0.0156 (11)
C20	0.0405 (12)	0.0339 (11)	0.0467 (13)	−0.0007 (9)	−0.0088 (10)	−0.0090 (10)
C21	0.0489 (14)	0.0408 (13)	0.0574 (16)	−0.0077 (11)	−0.0114 (12)	−0.0052 (11)
C22	0.0556 (16)	0.0552 (16)	0.0614 (17)	−0.0051 (13)	−0.0195 (13)	−0.0109 (13)
C23	0.0593 (16)	0.0433 (14)	0.0470 (15)	0.0084 (12)	−0.0148 (12)	−0.0099 (11)
C24	0.0685 (18)	0.0326 (12)	0.0525 (15)	−0.0043 (12)	−0.0093 (13)	−0.0040 (11)
C25	0.0506 (14)	0.0362 (12)	0.0555 (15)	−0.0057 (11)	−0.0125 (12)	−0.0085 (11)
N1	0.0386 (10)	0.0305 (9)	0.0398 (10)	−0.0009 (8)	−0.0004 (8)	−0.0074 (8)
O1	0.0599 (11)	0.0312 (8)	0.0680 (12)	−0.0069 (8)	0.0004 (9)	−0.0150 (8)
O2	0.0350 (8)	0.0574 (10)	0.0583 (11)	−0.0074 (7)	−0.0119 (8)	−0.0239 (8)
O3	0.0574 (11)	0.0555 (11)	0.0474 (10)	0.0035 (9)	−0.0069 (8)	−0.0232 (9)
O4	0.0674 (13)	0.0700 (13)	0.0780 (12)	0.0113 (10)	−0.0090 (11)	−0.0405 (11)
S1	0.0357 (3)	0.0335 (3)	0.0481 (3)	−0.0054 (2)	−0.0026 (2)	−0.0150 (2)
Cl1	0.0999 (6)	0.0621 (5)	0.0691 (5)	0.0133 (4)	−0.0379 (5)	−0.0014 (4)
Cl2	0.0858 (6)	0.0561 (4)	0.1016 (6)	−0.0308 (4)	−0.0391 (5)	−0.0005 (4)

### Geometric parameters (Å, º)

**Table:**

C1—C2	1.373 (3)	C14—O3	1.340 (3)
C1—C6	1.383 (3)	C14—C15	1.401 (3)
C1—S1	1.744 (2)	C15—C16	1.411 (3)
C2—C3	1.382 (3)	C15—C19	1.487 (3)
C2—H2	0.9300	C16—C17	1.380 (3)
C3—C4	1.370 (4)	C16—C20	1.488 (3)
C3—H3	0.9300	C17—C18	1.391 (3)
C4—C5	1.370 (4)	C17—H17	0.9300
C4—H4	0.9300	C18—N1	1.414 (3)
C5—C6	1.371 (3)	C19—O4	1.201 (3)
C5—H5	0.9300	C19—H19	0.9300
C6—H6	0.9300	C20—C25	1.383 (3)
C7—C8	1.386 (3)	C20—C21	1.383 (3)
C7—C12	1.392 (3)	C21—C22	1.378 (3)
C7—N1	1.431 (3)	C21—H21	0.9300
C8—C9	1.371 (4)	C22—C23	1.367 (4)
C8—H8	0.9300	C22—H22	0.9300
C9—C10	1.378 (4)	C23—C24	1.367 (4)
C9—H9	0.9300	C23—Cl1	1.731 (2)
C10—C11	1.376 (3)	C24—C25	1.379 (3)
C10—H10	0.9300	C24—H24	0.9300
C11—C12	1.386 (3)	C25—Cl2	1.730 (3)
C11—H11	0.9300	N1—S1	1.6557 (19)
C12—C13	1.439 (3)	O1—S1	1.4145 (16)
C13—C18	1.389 (3)	O2—S1	1.4211 (17)
C13—C14	1.390 (3)	O3—H3A	0.826 (10)
			
C2—C1—C6	120.9 (2)	C14—C15—C19	118.8 (2)
C2—C1—S1	119.29 (17)	C16—C15—C19	121.1 (2)
C6—C1—S1	119.76 (17)	C17—C16—C15	120.9 (2)
C1—C2—C3	119.3 (2)	C17—C16—C20	119.0 (2)
C1—C2—H2	120.4	C15—C16—C20	119.98 (19)
C3—C2—H2	120.4	C16—C17—C18	117.7 (2)
C4—C3—C2	119.7 (2)	C16—C17—H17	121.2
C4—C3—H3	120.2	C18—C17—H17	121.2
C2—C3—H3	120.2	C13—C18—C17	122.80 (19)
C5—C4—C3	120.9 (2)	C13—C18—N1	107.83 (18)
C5—C4—H4	119.6	C17—C18—N1	129.4 (2)
C3—C4—H4	119.6	O4—C19—C15	122.3 (2)
C4—C5—C6	120.0 (2)	O4—C19—H19	118.9
C4—C5—H5	120.0	C15—C19—H19	118.9
C6—C5—H5	120.0	C25—C20—C21	116.9 (2)
C5—C6—C1	119.2 (2)	C25—C20—C16	123.7 (2)
C5—C6—H6	120.4	C21—C20—C16	119.4 (2)
C1—C6—H6	120.4	C22—C21—C20	122.2 (2)
C8—C7—C12	121.5 (2)	C22—C21—H21	118.9
C8—C7—N1	130.3 (2)	C20—C21—H21	118.9
C12—C7—N1	108.20 (18)	C23—C22—C21	118.6 (3)
C9—C8—C7	117.5 (2)	C23—C22—H22	120.7
C9—C8—H8	121.2	C21—C22—H22	120.7
C7—C8—H8	121.2	C22—C23—C24	121.5 (2)
C8—C9—C10	121.8 (2)	C22—C23—Cl1	119.5 (2)
C8—C9—H9	119.1	C24—C23—Cl1	119.0 (2)
C10—C9—H9	119.1	C23—C24—C25	118.7 (2)
C11—C10—C9	120.8 (2)	C23—C24—H24	120.7
C11—C10—H10	119.6	C25—C24—H24	120.7
C9—C10—H10	119.6	C24—C25—C20	122.1 (2)
C10—C11—C12	118.7 (2)	C24—C25—Cl2	117.76 (19)
C10—C11—H11	120.7	C20—C25—Cl2	120.19 (19)
C12—C11—H11	120.7	C18—N1—C7	107.83 (17)
C11—C12—C7	119.8 (2)	C18—N1—S1	125.70 (15)
C11—C12—C13	132.9 (2)	C7—N1—S1	125.35 (14)
C7—C12—C13	107.26 (19)	C14—O3—H3A	106 (2)
C18—C13—C14	119.19 (19)	O1—S1—O2	120.49 (11)
C18—C13—C12	108.76 (18)	O1—S1—N1	106.41 (10)
C14—C13—C12	132.1 (2)	O2—S1—N1	106.55 (10)
O3—C14—C13	118.5 (2)	O1—S1—C1	108.93 (10)
O3—C14—C15	122.2 (2)	O2—S1—C1	108.09 (10)
C13—C14—C15	119.2 (2)	N1—S1—C1	105.36 (10)
C14—C15—C16	120.03 (19)		
			
C6—C1—C2—C3	0.8 (4)	C16—C17—C18—C13	−2.0 (3)
S1—C1—C2—C3	−177.4 (2)	C16—C17—C18—N1	179.1 (2)
C1—C2—C3—C4	−0.7 (4)	C14—C15—C19—O4	1.2 (4)
C2—C3—C4—C5	0.0 (4)	C16—C15—C19—O4	179.0 (2)
C3—C4—C5—C6	0.6 (4)	C17—C16—C20—C25	−111.3 (3)
C4—C5—C6—C1	−0.6 (4)	C15—C16—C20—C25	71.2 (3)
C2—C1—C6—C5	−0.1 (3)	C17—C16—C20—C21	69.8 (3)
S1—C1—C6—C5	177.99 (18)	C15—C16—C20—C21	−107.7 (3)
C12—C7—C8—C9	−1.6 (3)	C25—C20—C21—C22	−2.0 (4)
N1—C7—C8—C9	178.3 (2)	C16—C20—C21—C22	176.9 (2)
C7—C8—C9—C10	0.6 (4)	C20—C21—C22—C23	0.4 (4)
C8—C9—C10—C11	1.1 (4)	C21—C22—C23—C24	1.4 (4)
C9—C10—C11—C12	−1.8 (4)	C21—C22—C23—Cl1	−178.6 (2)
C10—C11—C12—C7	0.9 (3)	C22—C23—C24—C25	−1.3 (4)
C10—C11—C12—C13	−176.5 (2)	Cl1—C23—C24—C25	178.6 (2)
C8—C7—C12—C11	0.8 (3)	C23—C24—C25—C20	−0.5 (4)
N1—C7—C12—C11	−179.09 (19)	C23—C24—C25—Cl2	179.5 (2)
C8—C7—C12—C13	178.8 (2)	C21—C20—C25—C24	2.1 (4)
N1—C7—C12—C13	−1.1 (2)	C16—C20—C25—C24	−176.8 (2)
C11—C12—C13—C18	176.4 (2)	C21—C20—C25—Cl2	−177.94 (19)
C7—C12—C13—C18	−1.2 (2)	C16—C20—C25—Cl2	3.2 (3)
C11—C12—C13—C14	−3.0 (4)	C13—C18—N1—C7	−3.7 (2)
C7—C12—C13—C14	179.4 (2)	C17—C18—N1—C7	175.4 (2)
C18—C13—C14—O3	177.4 (2)	C13—C18—N1—S1	−172.07 (15)
C12—C13—C14—O3	−3.2 (4)	C17—C18—N1—S1	7.0 (3)
C18—C13—C14—C15	−1.6 (3)	C8—C7—N1—C18	−176.9 (2)
C12—C13—C14—C15	177.8 (2)	C12—C7—N1—C18	3.0 (2)
O3—C14—C15—C16	179.6 (2)	C8—C7—N1—S1	−8.5 (3)
C13—C14—C15—C16	−1.4 (3)	C12—C7—N1—S1	171.39 (15)
O3—C14—C15—C19	−2.6 (3)	C18—N1—S1—O1	−162.99 (18)
C13—C14—C15—C19	176.4 (2)	C7—N1—S1—O1	30.6 (2)
C14—C15—C16—C17	2.9 (3)	C18—N1—S1—O2	−33.2 (2)
C19—C15—C16—C17	−174.9 (2)	C7—N1—S1—O2	160.35 (17)
C14—C15—C16—C20	−179.7 (2)	C18—N1—S1—C1	81.44 (19)
C19—C15—C16—C20	2.5 (3)	C7—N1—S1—C1	−84.97 (19)
C15—C16—C17—C18	−1.2 (3)	C2—C1—S1—O1	135.88 (19)
C20—C16—C17—C18	−178.66 (19)	C6—C1—S1—O1	−42.3 (2)
C14—C13—C18—C17	3.4 (3)	C2—C1—S1—O2	3.3 (2)
C12—C13—C18—C17	−176.1 (2)	C6—C1—S1—O2	−174.85 (17)
C14—C13—C18—N1	−177.47 (19)	C2—C1—S1—N1	−110.30 (19)
C12—C13—C18—N1	3.0 (2)	C6—C1—S1—N1	71.55 (19)

### Hydrogen-bond geometry (Å, º)

**Table:**

*D*—H···*A*	*D*—H	H···*A*	*D*···*A*	*D*—H···*A*
O3—H3*A*···O4	0.83 (1)	1.81 (2)	2.563 (3)	151 (3)
C2—H2···Cl1^i^	0.93	2.81	3.412 (2)	123
C5—H5···O2^ii^	0.93	2.49	3.184 (3)	131

Symmetry codes: (i) −*x*+1, −*y*+2, −*z*+1; (ii) *x*+1, *y*, *z*.

## References

[bb1] Allen, F. H., Kennard, O., Watson, D. G., Brammer, L., Orpen, A. G. & Taylor, R. (1987). *J. Chem. Soc. Perkin. Trans. 2*, pp. S1–19.

[bb2] Bassindale, A. (1984). *The Third Dimension in Organic Chemistry*, ch. 1, p. 11. New York: John Wiley and Sons.

[bb3] Bernstein, J., Davis, R. E., Shimoni, L. & Chang, N.-L. (1995). *Angew. Chem. Int. Ed. Engl.* **34**, 1555–1573.

[bb4] Bruker (2004). *APEX2* and *SAINT*. Bruker AXS Inc., Madison, Wisconsin, USA.

[bb5] Chakkaravarthi, G., Dhayalan, V., Mohanakrishnan, A. K. & Manivannan, V. (2008). *Acta Cryst.* E**64**, o1667–o1668. 10.1107/S1600536808024380 PMC2960676 21201660

[bb6] Chakkaravarthi, G., Marx, A., Dhayalan, V., Mohanakrishnan, A. K. & Manivannan, V. (2009). *Acta Cryst.* E**65**, o464–o465. 10.1107/S1600536809003493 PMC2968599 21582136

[bb7] Chakraborty, D. P., Barman, B. K. & Bose, P. K. (1965). *Tetrahedron*, **21**, 681–685.

[bb8] Chakraborty, D. P., Bhattacharyya, P., Roy, S., Bhattacharyya, S. P. & Biswas, A. K. (1978). *Phytochemistry*, **17**, 834–835.

[bb14] Groom, C. R. & Allen, F. H. (2014). *Angew. Chem. Int. Ed.* **53**, 662–671. 10.1002/anie.201306438 24382699

[bb9] Ramsewak, R. S., Nair, M. G., Strasburg, G. M., DeWitt, D. L. & Nitiss, J. L. (1999). *J. Agric. Food Chem.* **47**, 444–447. 10.1021/jf9805808 10563914

[bb10] Sheldrick, G. M. (1996). *SADABS*. University of Göttingen, Germany.

[bb11] Sheldrick, G. M. (2008). *Acta Cryst.* A**64**, 112–122. 10.1107/S0108767307043930 18156677

[bb12] Spek, A. L. (2009). *Acta Cryst.* D**65**, 148–155. 10.1107/S090744490804362X PMC2631630 19171970

[bb13] Tachibana, Y., Kikuzaki, H., Lajis, N. H. & Nakatani, N. (2001). *J. Agric. Food Chem.* **49**, 5589–5594. 10.1021/jf010621r 11714364

